# Qualitative and quantitative evaluation of diabetic choroidopathy using ultra-widefield indocyanine green angiography

**DOI:** 10.1038/s41598-023-29216-5

**Published:** 2023-02-13

**Authors:** Sang Uk Choi, Yoon Jeon Kim, Joo Yong Lee, Junyeop Lee, Young Hee Yoon

**Affiliations:** 1 Department of Ophthalmology, Chung-Ang University Hospital, Chung-Ang University, College of Medicine, Seoul, South Korea; 2 Department of Ophthalmology, Asan Medical Center, University of Ulsan, College of Medicine, 88 Olympic-ro 43-gil, Songpa-gu, Seoul, 05505 Korea; 3 Asan Diabetes Center, Asan Medical Center, Seoul, South Korea

**Keywords:** Eye manifestations, Retinal diseases

## Abstract

To investigate angiographic characteristic features of diabetic choroidopathy, as well as choroidal vascular density (CVD) and fractal dimension (CFD) in diabetic eyes and controls using ultra-widefield (UWF) indocyanine green angiography (ICGA). All patients underwent UWF fluorescein angiography and ICGA. Using imageJ software, CVD and CFD was analyzed. SFCT was assessed using spectral-domain optical coherence tomography. The image parameters were compared based on the DR stage and the presence of diabetic macular edema (DME). One-hundred six eyes from 63 patients (59.11 ± 16.31 years; male [%]: 23 [36.5%]) were included in the DM group, and 40 eyes from 22 subjects were included in the control group. The DM group had a mean age of 59.11 ± 16.31 years and a mean HbA1c percentage of 7.72 ± 1.28%. The most common ICGA findings of DC were choroidal hyperpermeability (57.5%), hypofluorescent spots (48.1%). Salt and pepper pattern (19.8%), inverted inflow phenomenon (3.8%), choroidal arterial tortuosity (24.5%), and late choroidal non-perfusion (6.6%) were more common in advanced DR. The CVD, CFD, and SFCT increased as the DR severity progressed. The DME group had a significantly higher CFD and SFCT than the non-DME group (*P* < 0.001 and *P* = 0.019, respectively). The qualitative and quantitative UWF ICGA image analysis revealed that choroidal blood vessels became dilated, complex, and hyperpermeable as the DR progressed. These features of diabetic choroidopathy (DC) were more severe in eyes with DME than the non-DME eyes.

## Introduction

Diabetes mellitus (DM) is a chronic metabolic disorder characterized by long-term systemic microvascular and macrovascular complications^1,2^. Diabetic retinopathy (DR) is the most prevalent and serious microvascular disease induced by chronic hyperglycemia in DM^3^. This is because the retina has a densely distributed network of blood vessels to meet the high metabolic demand of photoreceptor cells. The choroid, which is located outside the retina, is a unique tissue that is almost made up of blood vessels. It has the most blood flow of any tissue in the human body^4^. Previous histopathologic studies demonstrated that choriocapillaris degeneration in DM^5–7^.

Diabetic choroidopathy (DC) was previously characterized by choriocapillaris (CC) dropout, luminal constriction, and large and intermediate blood vessel tortuosity^5,6^. Diabetes caused degeneration of choroidal pericytes and affects CC structure and function^8–10^. Recent studies using optical coherence tomography (OCT) angiography indicated that CC loss in DM is highly related to photoreceptor degradation^11^. Meanwhile, the studies of DC using OCT have focused on subfoveal choroidal thickness, which remains disputed because both thickening and thinning have been detected^12,13^. In addition, several studies are limited because they did not take into account the history of ocular treatments and ophthalmologic interventions, such as panretinal photocoagulation or intravitreal anti-vascular endothelial growth factor (VEGF), which clearly have a substantial effect on choroidal thickness^14–17^.

Indocyanine green angiography (ICGA) is a clinically useful imaging technique for assessing the vascular structure of the deep choroid through the retinal tissue^18^. Previous ICGA image studies show choroidal vascular insufficiency in diabetic patients^19,20^. DC displays both hypofluorescent and hyperfluorescent spots in ICGA^19^. In addition, poor diabetic control was associated “salt and pepper” pattern in non-proliferative diabetic retinopathy (NPDR)^20^. Recent advances in ultra-widefield (UWF) imaging expand the ICGA’s applicability to evaluate the peripheral vasculature in various retinal and choroidal diseases^21–24^. Clinical features of diabetic choroidopathy on ICGA were not yet evaluated using UWF imaging and thus it is needed to be characterized according to the severity of DR. In this study, we analyzed the UWF ICGA findings and association with the choroidal thickness in OCT images to identify choroidal characteristics in ophthalmologically treatment-naïve DM patients.

## Results

In this study, 106 eyes from 63 patients (mean age ± SD: 59.11 ± 16.31 years; male [%]: 23 [36.5%]) were included in the DM group, and 40 eyes from 22 subjects were included in the control group. The baseline characteristics of the studied groups are summarized in Table 1. At the time of enrollment, the DM group had a relatively well-controlled status (mean HbA1c level: 7.72 ± 1.28%). In addition, the average duration of DM was 7.39 ± 1.74 years. There were no significant differences in age, gender, or intraocular pressure between the DM and control groups (Table 1). Among 106 DM eyes, 18 (17.0%) had no DR, 32 (30.2%) had mild NPDR, 10 (9.4%) had moderate NPDR, 16 (15.1%) had severe NPDR, 30 (28.3%) had PDR, and 18 had diabetic macular edema (DME).

**Table 1:** Baseline characteristics.

	DM group	Control group	*P*-value
Patients (n)	63	22	
Age	59.11 ± 16.31	58.71 ± 7.97	0.597^a^
Female/male	40:23	12:10	0.443^b^
HTN (n)	33 (51.1%)	10 (45.4%)	0.576^b^
HbA1c (%)	7.72 ± 1.28	NA	
DM duration (years)	7.39 ± 1.74	NA	
Eye (n)	106	40	
VA (LogMAR)	0.09 ± 0.22	0	< 0.010*
IOP (mmHg)	15.46 ± 3.53	15.16 ± 3.43	0.719*
DR stages (eye, n, %)		NA	
No DR	18 (16.9%)		
Mild NPDR	32 (30.2%)		
Moderate NPDR	10 (9.4%)		
Severe NPDR	16 (15.0%)		
PDR	30 (28.3%)		
DME (n, %)	18 (16.9%)	NA	

^a^Independent t-test.

^b^Chi-squared test.

HTN, hypertension; DM, diabetes mellitus; VA, visual acuity; IOP, intraocular pressure; DR, diabetic retinopathy; NPDR, non-proliferative diabetic retinopathy; PDR, proliferative diabetic retinopathy; DME, diabetic macular edema.

**Figure 1:**
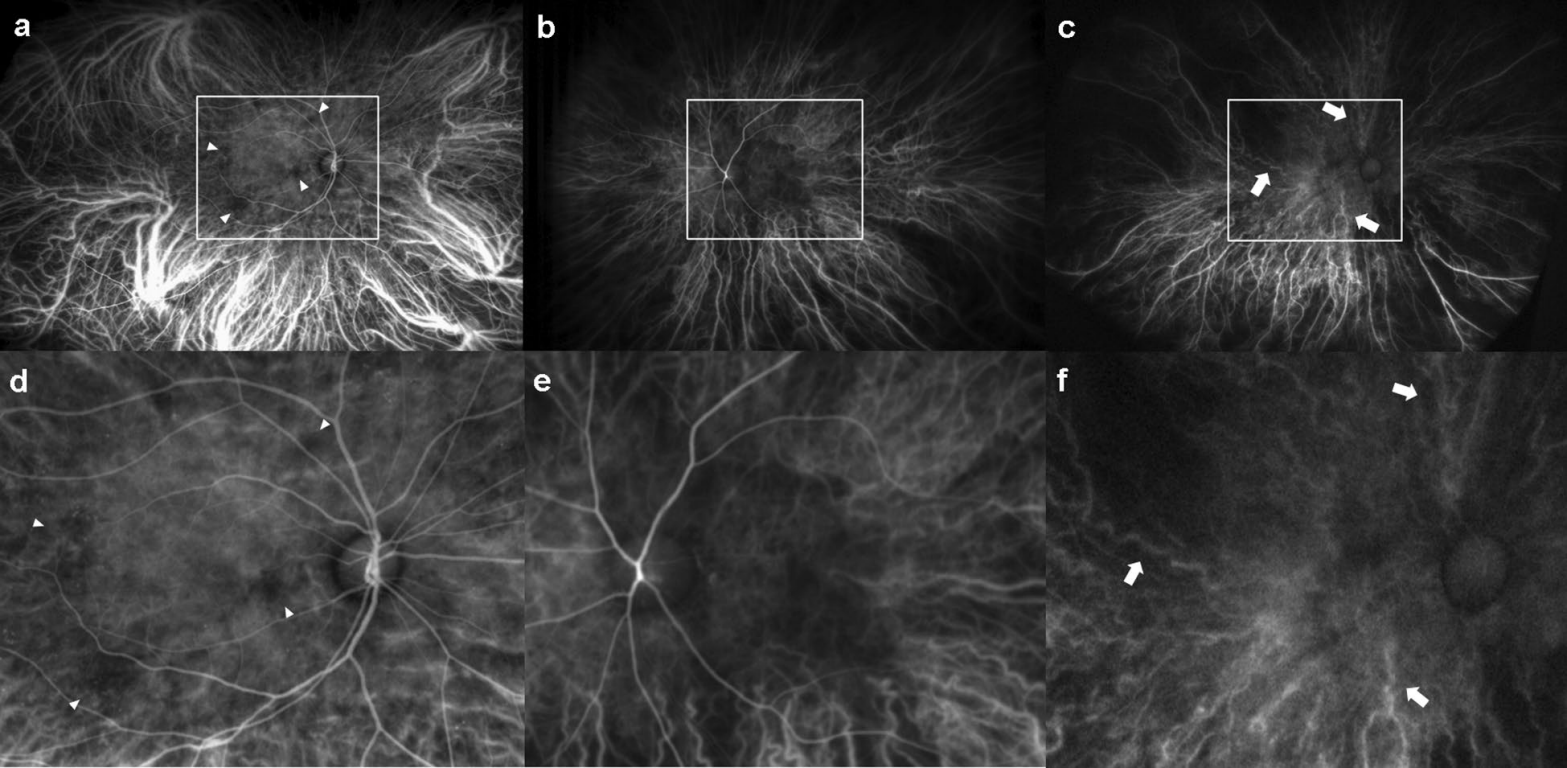
Early phase indocyanine green angiography (ICGA) findings of diabetic choroidopathy. The top row includes the original ICGA image. The bottom row includes the posterior pole enlarged image (the box in the upper row). (**a**, **d**) Hypofluorescent spots are detected in the early phase, indicating delay or ischemia in choriocapillaris filling (arrowhead). (**b**, **e**) Inverted inflow phenomenon with retinal arteries filling before choroidal perfusion. Choroidal blood flow is delayed due to increased choroidal vascular resistance. (**c**, **f**) A diabetic patient shows severe non-proliferative diabetic retinopathy with tortuous and corkscrew features of the choroidal artery in very early ICGA (arrow).

**Figure 2:**
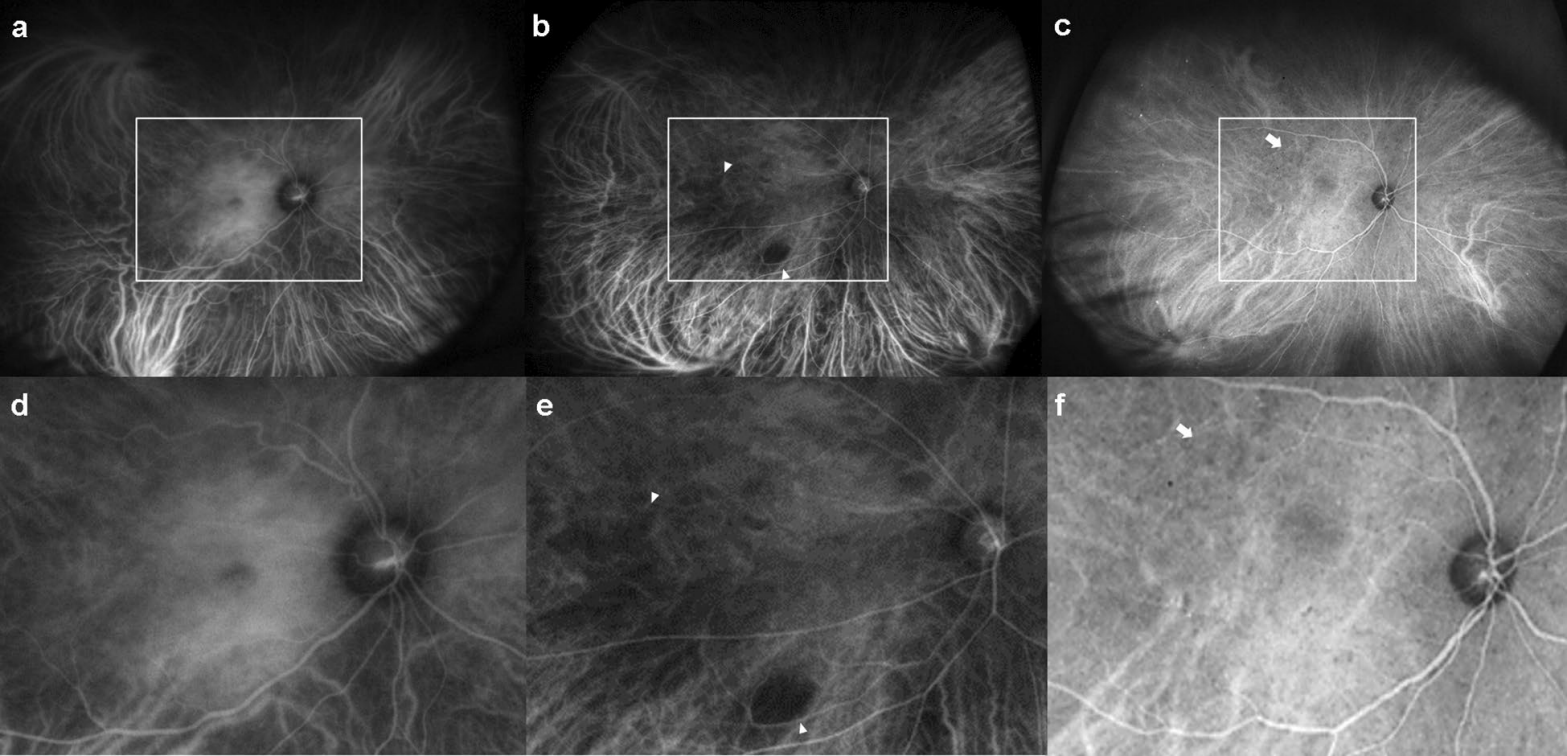
Late phase indocyanine green angiography (ICGA) findings of diabetic choroidopathy. The top row includes the original ICGA image. The bottom row includes the posterior pole enlarged image (the box in the upper row). (**a**, **d**) Choroidal hyperpermeability with a diffuse hyperfluorescence at the macula or around the optic nerve in the late phase of ICGA is caused by the diffusion of indocyanine green molecules from the choriocapillaris to interstitial tissues. (**b**, **e**) Late choroidal non-perfusion with choroidal vascular non-perfusion regions in the late phase of ICGA secondary to choroidal vascular occlusion (arrowhead). (**c**, **f**) “Salt and pepper” pattern with lobular spotty hyperfluorescence and hypofluorescence in the posterior pole in the late phase of an ICGA microinfarct at the choriocapillaris or fluorescence blocking by inflammatory cellular aggregates (arrow).

**Figure 3:**
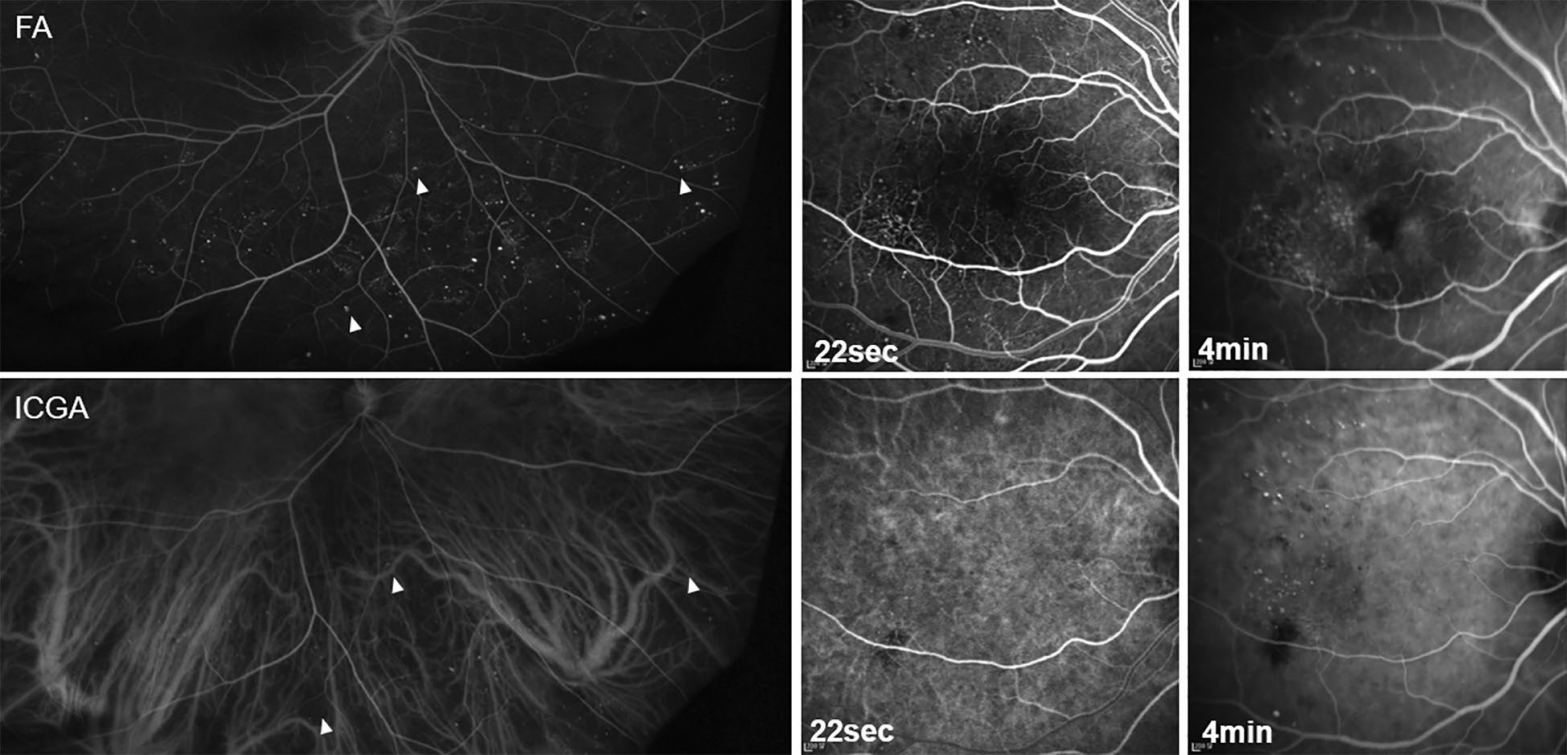
Fluorescein angiography (FA, upper row) and indocyanine green angiography (ICGA, lower row) of a patient with severe non-proliferative diabetic retinopathy. There were several microaneurysms (MAs) that were only observed in the FA image and not in the ICGA image (arrowhead, left column). The number of visible MAs was higher in the FA than in the ICGA (center and right column). MAs appearing in the ICGA were more clearly observed in the late phase rather than early phase (bottom center and bottom right). There was no prominent leakage in the MA in the late phase of ICGA (bottom, right).

The most prevalent early and late qualitative DC findings on UWF ICGA were hypofluorescent spots (Fig. 1a,d) and choroidal hyperpermeability (Fig. 2a,d), accounting for 48.1% and 57.5% of the total DM group, respectively (Table 2). However, these two UWF ICGA findings were present in 7.5% and 10% of the control group, respectively (Table 2). The salt and pepper pattern, inverted inflow phenomenon, late choroidal non-perfusion, and choroidal artery tortuosity were only detected in the DM group but not in the control group (Figs. 1b,c,e,f, 2b,c,e,f, respectively, Table 2). In advanced NPDR and PDR, inverted inflow phenomenon, late choroidal non-perfusion, and choroidal arterial tortuosity were more prevalent (Table 2). Microaneurysms (MA) were detected in FA and ICGA images of the same phase (Fig. 3). However, there was no MA, which was not visible in the FA image and only visible in the ICGA image. (Fig. 3). In addition, MA appeared on ICGA relatively later and did not show prominent leakage, while MA on FA appeared earlier and showed prominent leakage later (Fig. 3).

**Table 2:** Quantitative analysis according to diabetic retinopathy stages.

	DM with no DR (n = 18)	Mild NPDR (n = 32)	Moderate NPDR (n = 10)	Severe NPDR (n = 16)	PDR (n = 30)	DR eyes (%) (n = 106)	Control eyes (%) (n = 40)
Choroidal hyperpermeability	9	17	6	10	19	61 (57.5)	4 (10)
Hypofluorescent spots	7	14	5	8	17	51 (48.1)	3 (7.5)
“Salt and pepper” pattern	0	6	4	2	9	21 (19.8)	0
Inverted inflow phenomenon	0	0	0	0	4	4 (3.8)	0
Late choroidal non-perfusion	0	0	2	1	4	7 (6.6)	0
Choroidal arterial tortuosity	1	1	3	6	15	26 (24.5)	0

DM, diabetes mellitus; DR, diabetic retinopathy; NPDR, non-proliferative diabetic retinopathy; PDR, proliferative diabetic retinopathy.

**Figure 4:**
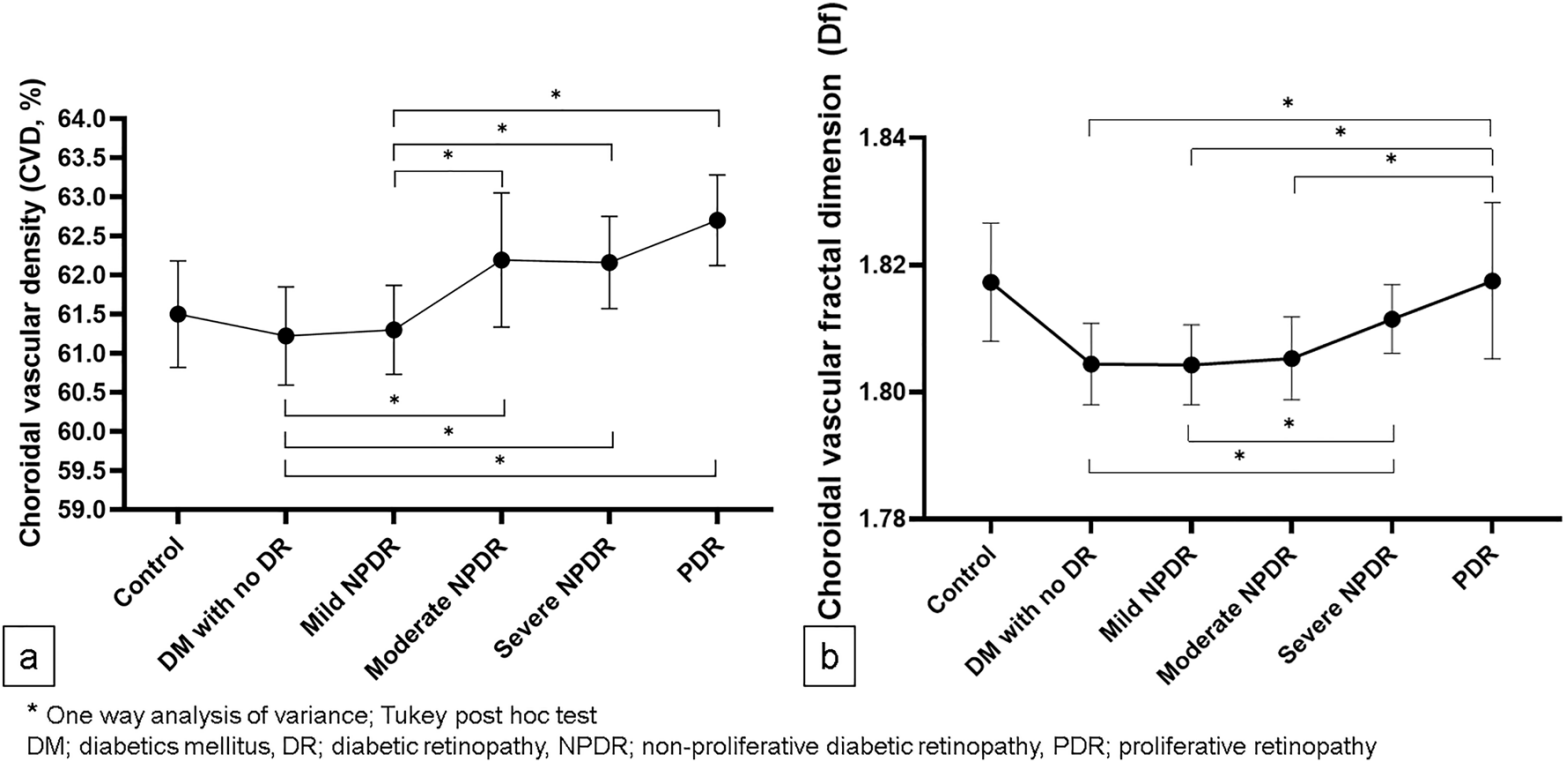
Quantitative analysis of indocyanine green angiography results by diabetic retinopathy stage. (**a**) Choroidal vascular density (CVD, %) was significantly lower in the no diabetic retinopathy and mild non-proliferative diabetic retinopathy (NPDR) groups than in the moderate NPDR, severe NPDR, and proliferative retinopathy (PDR) groups. (**b**) Choroidal vascular fractal dimension (CFD) was significantly higher in the severe NPDR group than in the no diabetic retinopathy and mild NPDR groups. The PDR group showed a significantly higher CFD than the no diabetic retinopathy, mild NPDR, and moderate NPDR groups.

According to the DR severity, CVD and CFD had a gradual upward trend (Fig. 4a,b, respectively). DM with no DR had a significantly lower mean CVD than moderate NPDR, severe NPDR, or PDR (Fig. 4a, 61.22 ± 0.63, 62.19 ± 0.86, 62.16 ± 0.59, and 62.70 ± 0.58; *P* = 0.009, *P* < 0.001, and *P* < 0.001, respectively). In addition, mild NPDR had a significantly lower mean CVD than more severe DR (Fig. 4a, 61.30 ± 0.57, 62.19 ± 0.86, 62.16 ± 0.59, and 62.70 ± 0.58; *P* = 0.008, *P* < 0.001, and *P* < 0.001, respectively). On the other hand, severe NPDR had a significantly higher CFD than DM with no DR or mild NPDR (Fig. 4b, 1.8115 ± 0.0054, 1.8044 ± 0.0064, and 1.8043 ± 0.0063; *P* = 0.040, and *P* = 0.005, respectively). In addition, PDR had a significantly higher CFD than DM with no DR, mild NPDR, or moderate NPDR (Fig. 4b, 1.8175 ± 0.0123, 1.8044 ± 0.0064, 1.8043 ± 0.0063, and 1.8053 ± 0.0065; *P* < 0.001, *P* < 0.001, and *P* = 0.002, respectively). In DR group, all patients with DME had severe NPDR and PDR. Subgroup analysis of CVD and CFD in severe NPDR and PDR groups based on the presence of DME revealed that the DME group has a significantly higher CFD than the non-DME group, while, CVD had no significant difference between the groups (Table 3).

**Table 3:** Quantitative analysis of severe non-proliferative retinopathy and proliferative retinopathy groups based on the presence of diabetic macular edema (DME).

	Non-DME group (n = 28)	DME group (n = 18)	*P*-value^a^
Choroidal vascular density (%)	62.41 ± 0.62	62.32 ± 0.68	0.658
Choroidal vascular fractal dimension	1.8110 ± 0.0073	1.8183 ± 0.0101	0.008

^a^Mann–Whitney U test.

**Figure 5:**
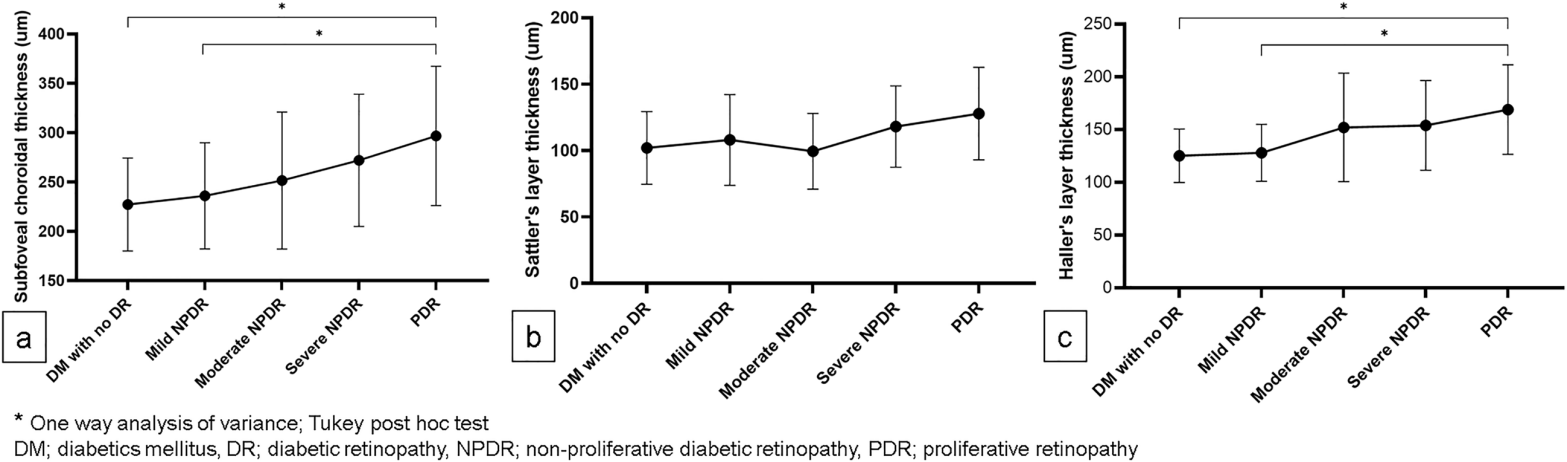
Subfoveal choroidal thickness (SFCT) by diabetic retinopathy stage. (**a**) The total SFCT was significantly lower in the no diabetic retinopathy and mild non-proliferative diabetic retinopathy (NPDR) groups than in the proliferative retinopathy (PDR) group. (**b**) Sattler’s layer thickness showed no significant difference among the groups. (**c**) Haller’s layer thickness was significantly lower in the no diabetic retinopathy and mild NPDR groups than in the PDR group.

Similar to CVD and CFD, SFCT showed a tendency to increase as diabetic retinopathy worsened. (Fig. 5a). PDR had a significantly higher SFCT than DM with no DR or mild NPDR (Fig. 4a, 296.74 ± 70.59, 227.20 ± 47.07, and 235.96 ± 53.85; *P* = 0.020 and *P* = 0.017, respectively). Sattler’s layer thickness tended to increase according to the DR stage with no significant difference among the groups (Fig. 5b). On the other hand, PDR had a significantly higher Haller’s layer thickness than DM with no DR or mild NPDR (Fig. 5c, 168.95 ± 42.41, 125.24 ± 25.38, and 127.94 ± 26.98; *P* = 0.014 and *P* = 0.005, respectively). Subgroup analysis of SFCT in severe NPDR and PDR groups based on the presence of DME revealed that the DME group has thicker SFCT and Haller’s layer thickness than the non-DME group (Table 4, *P* = 0.019 and *P* = 0.009, respectively).

**Table 4:** Subfoveal choroidal thickness based on the presence of diabetic macular edema (DME).

	Non-DME group (n = 28)	DME group (n = 18)	*P* valuea
Sub-foveal choroidal thickness	260.65 ± 66.39	311.72 ± 61.81	0.019
Sattler’s layer	116.02 ± 35.91	130.24 ± 24.83	0.214
Haller’s layer	144.62 ± 36.38	181.48 ± 42.46	0.009

^a^Mann–Whitney U test.

## Discussion

In this study, we investigated the DC characteristics and quantitatively evaluated the UWF ICGA images in patients with DM. To the best of our knowledge, this is the first UWF ICGA qualitative and quantitative analysis according to DR severity. We demonstrated that hypofluorescent spots and choroidal hyperpermeability were frequent UWF ICGA findings in patients with DC, and the “salt and pepper” pattern, choroidal artery tortuosity, late choroidal non-perfusion, inverted inflow phenomenon were more common in patients with advanced DR. Notably, quantitative image analysis revealed that CVD and CFD were increased according to the DR stage, which was consistent with the increase in SFCT.

Diabetic choroidopathy was first described by Hidayat and Fine, which included choriocapillaris dropout, basement membrane thickening, and choroidal neovascularization in patients with advanced DM on light microscopy^5^. Another histopathologic study, reported the diabetic choroid was characterized by choriocapillaris degeneration, which included increased alkaline phosphatase activity and extravascular migration of polymorphonuclear leukocytes^7^. These histopathologic studies revealed that DM could affect the choroidal circulation.

With the development of ICGA imaging technology, there have been studies that identified and defined DM.

Weinberger and Gaton were the first ones to report the ICGA findings of patients with NPDR^20^. They employed conventional 50-degree ICGA imaging and reported a late phase “salt and pepper” pattern and hypofluorescence spots, which is the characteristic pattern of choroidal changes in NPDR. In our study, we discovered a “salt and pepper” pattern is more frequent in advanced NPDR and PDR eyes than in early NPDR or no DR eyes (Table 2). The “salt and pepper” pattern of selective CC filling indicates focal choroidal ischemia^20^. Therefore, DR severity may exacerbate choroidal vascular damage. Only 60–80% of the small fluorescein molecules are bound to plasma proteins and thus leak through the fenestrated choriocapillaris, causing “background staining.” Unlike fluorescein, up to 98% of ICG molecules are bound to plasma proteins and do not leak through the fenestrated choriocapillaris. Therefore, the hyperfluorescence appearance of ICGA could be interpreted as a breakdown of the CC or a small choroidal vessel and subsequent leakage of the ICG molecule. Meanwhile, in control eyes, choroidal hyperpermeability and hypofluorescent spots were detected (Table 2). Thus, choroidal vascular leakage on ICGA is not a characteristic feature of DC.

In the early phases of ICGA, the choroidal artery was detected as a radial linear contrast enhancement pointing posteriorly (Fig. 1b,c). The choroidal artery exhibits corkscrew-like tortuosity around the posterior pole area, particularly in advanced NPDR and PDR eyes (Table 2, Fig. 1c,f). In PDR eyes, an inverted inflow phenomenon was observed, in which retinal arteries started to fill before choroidal perfusion (Fig. 1b,e). Previous studies employing doppler imaging indicated that as DR severity increased, choroidal blood flow decreased and choroidal vascular resistance increased^25–27^. Thus, these two ICGA findings could be attributed to increased choroidal vascular resistance. This has the potential to be used as a characteristic qualitative ICGA finding of DC. However, due to the cross-sectional design of this study, we could not determine whether these findings appear in a chronological order with DC progression.

MA is defined as a saccular expansion of the capillary wall, which is primarily detected in the retina but also in other organs such as the kidneys and hearts of diabetic patients. These MAs cause endothelial hypercellularity and selective pericyte loss, resulting in vascular wall weakness^28^. An early ICGA imaging study detected MA; however, it did not ensure that it was true choroidal MA or early choroidal neovascularization as described in the pathological examination^20^. In our study, we confirmed that all MAs observed in ICGA were also apparent in FA (Fig. 3). However, the number of MA in ICGA was lower than that in FA (Fig. 3). This is thought to be due to the size of the MA being large enough for the protein-bound indocyanine green molecule to pass through and then be detected by ICGA^20^.

Previous SFCT or subfoveal CVI studies using OCT imaging only show a limited portion of the choroid in the posterior pole. The findings of OCT studies remain controversial. Both choroidal thinning and thickening have been reported^13,29–31^. The ocular therapy for DR is an important factor to consider when interpreting these findings. Both intravitreal anti-VEGF injection and panretinal photocoagulation induce a choroidal thinning^32^. This consideration could be avoided only treatment-naïve patients with DM were targeted. According to the DR severity, our findings demonstrated a significant increase in CVD and SFCT (Figs. 4a and 5a). Interestingly, only Haller’s layer, not Sattler’s layer, shows significant thickening as DR severity increases in the SFCT sub-layer analysis (Fig. 5c,b, respectively). According to these findings, choroidal vessels dilated as DC progressed, which is thought to be due to the dilation of Haller’s layer. Given that the ICGA image did not show CC vasculature, dilation of choroidal vasculature in DC was mainly responsible for the change in medium- to large-sized choroidal vessels. Perivascular cells express different morphologies and functions along with vascular trees^33^. Veins are covered by vascular smooth muscle cells (VSMC), which are substantially less dense than arteries^33^. In addition, in recent studies, veins were found to exhibit NG2 and platelet-derived growth factor receptor beta (PDGFRβ) expressing pericytes^34,35^. In hyperglycemia, protein kinase C-delta (PKC-δ) upregulation inhibits the PDGF/PDGFRβ-Αkt pathway, reducing pericyte survival and inducing pericyte apoptosis^36,37^. Therefore, reduced pericyte coverage or changes in pericyte characters may cause changes in CVD and choroidal thickness in DC.

A fractal is a geometric pattern that allows the description of objects that branch repeatedly^38^. The fractal dimension (Df) represents the complexity of the branching pattern of the vasculature, and it is a ratio with no counting units. The reduction in retinal vascular Df indicates early vascular changes in DR^39,40^. In this study, CVD and CFD were significantly higher in advanced DR eyes than in early or no DR eyes (Fig. 4b). Given that no choroidal neovascularization was observed in the qualitative evaluation, the simultaneous increase in CVD and CFD indicates that choroidal vessels dilate and tortuosity increases at the same time. Based on the findings of a Doppler hemodynamic study, the increase in CFD can be interpreted as a cause or effect of an increase in choroidal vascular resistance^26^. The gradual increase in CFD based on DR stages and choroidal artery tortuosity in advanced DR (Table 2) indicates that choroidal vascular resistance increases with DR severity degree. The choroidal vessels have VSMC coverage that is denser in choroidal arteries^33^. In DM, hyperglycemia inhibits VSMC apoptosis while enhancing VSMC proliferation^41,42^. Transforming growth factor-beta (TGFβ) is upregulated in diabetic endothelial cells, promoting Smad2/3 signaling in perivascular cells and mesenchymal cell differentiation into VSMC^43,44^. TGFβ1 also induces VSMC phenotypic switch to contractile phenotype^45^. These microvascular mural cell phenotypic changes could be explained by a gradual increase in CFD by the DR stage and an increase in choroidal vascular resistance in DC.

DME was caused by a local effect of VEGF and pro-inflammatory cytokines synthesized by the retina^46^. Based on the presence or absence of DME, subgroup analysis revealed significantly high CFD, SFCT, and Haller’s layer thickness in the DME group. These findings demonstrate how localized retinal inflammation and VEGF activity in DR affect choroidal vasculature. Based on these findings, CFD with SFCT, particularly Haller’s layer thickness, could be used as a biomarker for treatment response monitoring of DME.

This study has several limitations. First, the retinal vasculature was not subtracted from the image analysis process. This may result in underestimation of fractal dimension and vascular density in advanced DR with retinal non-perfusion. Second, because images were used without stereographic projection, peripheral CVD may be overestimated. Third, because the UWF ICGA detects mainly medium- to large-sized choroidal vessels, choriocapillaris cannot be analyzed in this study. Fourth, renal function, which has a significant effect on choroidal thickness, was not considered. Finally, because the choroidal vascular disease is ambiguous, the possibility of co-existing choroidal diseases such as pachychoroid spectrum disease cannot be entirely excluded.

In conclusion, we revealed that diabetes induces choroidal vascular dilation and increases choroidal vascular complexity through UWF ICGA analysis. An advanced diabetic choroidopathy had a characteristic UWF ICGA pattern, including the “salt and pepper” pattern, inverted inflow phenomenon, late choroidal non-perfusion, and choroidal artery tortuosity, indicating choroidal inflammation and increased vascular resistance. Future longitudinal studies should focus on DC clinical staging and its therapeutic applications.

## Methods

### Study population

This cross-sectional, retrospective, observational study was conducted at the Ophthalmology Department of Asan Medical Center in the period between June 1, 2020, and February 28, 2021. The study protocol adhered to the principles of the Helsinki Declaration and was approved by the Institutional Review Board and Ethics Committee of the Asan Medical Center. A treatment-naïve patients with DM who underwent UWF ICGA were included in this study. The exclusion criteria were as follows: (1) age less than 18 years; (2) type 1 DM; (3) high myopia (spherical equivalent $\le -$6D); (4) retinal vascular disease other than DR; (5) history of posterior or intermediate uveitis, vitreous opacity, and any ocular pathology that could significantly impact imaging studies; and (6) intraocular surgery, excluding routine cataract surgery. All subjects underwent complete ophthalmic examinations, including best-corrected visual acuity, intraocular pressure, dilated fundus examination, spectral-domain OCT (SD-OCT, Spectralis: Heidelberg Engineering, Heidelberg, Germany) with enhanced depth imaging (EDI), UWF FA, and ICGA (Optos California: Optos plc, Dunfermline, United Kingdom). Diabetic retinopathy was graded according to Early Treatment Diabetic Retinopathy Study criteria^47,48^. Diabetic macular edema (DME) defined as central macular thickness $\ge$ 300 um based on SD-OCT^49^. In this study, control group were subjects without DM who visited a retina clinic to assess vitreous opacity, epiretinal membrane.

### UWF angiography image acquisition and analysis

UWF FA and ICGA were performed after obtaining informed consent from the patient. Following an intravenous injection of 5 mL of 10% sodium fluorescein, UWF FA was conducted. UWF ICGA was then performed 30 min after UWF FA, with an intravenous injection of 2 mL of 25 mg of indocyanine green., while choroidal vasculature was quantitatively analyzed using the early phase UWF ICGA images (1–2 min after dye injection). A selected image was converted to a JPG file and the choroidal vascular density (CVD) and the choroidal vascular fractal dimension (CFD) were analyzed using ImageJ version 1.53 h (National Institute of Health, Bethesda, MD, USA) modified methods from the previous reports (eFigure 1. supplementary appendix)^50,51^. Following analysis, CVD was first calculated. An oval region of interest (ROI) (3200 × 2400 pixel) centered on the fovea was then determined using the Image J oval ROI tool and confirmed by two retina specialists (S.U.C and J.L). The binarized image was then adjusted by Niblack’s auto local thresholding and converted to a red, green, and blue image, and the luminal area was estimated using the color threshold tool. The light pixels were defined as the luminal area, which was automatically calculated by pixel. For CFD, the same ROI, Niblack’s auto local thresholding was used, the binarized image was skeletonized, and the CFD was assessed using the Image J’s FracLac plug-in (https://imagej.nih.gov/ij/plugins/fraclac/fraclac.html).

### Subfoveal choroidal thickness measurement

The subfoveal choroidal thickness (SFCT), Sattler’s layer thickness, and Haller’s layer thickness were measured using an EDI SD-OCT image in the subfoveal region. The SFCT was defined as the vertical distance between the hyper-reflective line of Bruch’s membrane and the line connecting the outer margin of the large choroidal vessel layer. Haller’s layer was defined as the outer choroidal vessel layer composed of the choroidal large vessels^52^. Sattler’s layer was defined as a medium-sized vessel layer in which hypo-intense spaces were surrounded by a hyper-intensive stroma^52^. The vertical distance between the inner and outer margins of the large choroidal vessel layer was used to calculate the thickness of Haller’s layer. The thickness of Sattler’s layer was calculated by subtracting Haller’s layer thickness from the total choroidal thickness. Two independent masked graders (S.U.C and J.L) took measurements at a single point below the fovea using the built-in caliper tool (Heidelberg Eye Explorer, Version 1.9.10, Heidelberg Engineering).

### Data analysis

The qualitative data were presented in a descriptive manner by DR stages. The quantitative data (CVD and CFD) were compared between DR stages and the groups studied. All statistical analyses were performed using the Statistical Package for the Social Sciences, version 20 (IBM Co., Armonk, New York, USA). Continuous variables were presented as mean and standard deviation (SD), while categorical variables were presented as numbers (n) and percentages (%). A two-sided p-value of less than 0.05 was considered significant.

### Conference presentation

Presented at the 38th annual meeting of the American Society of Retina Specialists, 2021. San Antonio, United State of America.

## Supplementary Information

Supplementary Information.

## Data Availability

The datasets are available from the corresponding author upon reasonable request.
