# Author Correction: Qualitative and quantitative evaluation of diabetic choroidopathy using ultra-widefield indocyanine green angiography

**DOI:** 10.1038/s41598-023-30205-x

**Published:** 2023-02-28

**Authors:** Sang Uk Choi, Yoon Jeon Kim, Joo Yong Lee, Junyeop Lee, Young Hee Yoon

**Affiliations:** 1 Department of Ophthalmology, Chung-Ang University Hospital, Chung-Ang University, College of Medicine, Seoul, South Korea; 2 Department of Ophthalmology, Asan Medical Center, University of Ulsan, College of Medicine, 88 Olympic-ro 43-gil, Songpa-gu, Seoul, 05505 Korea; 3 Asan Diabetes Center, Asan Medical Center, Seoul, South Korea

Correction to: *Scientific Reports* 10.1038/s41598-023-29216-5, published online 13 February 2023

The Funding section in the original version of this Article was omitted.

The Funding section now reads: “This research was supported by Basic Science Research Program through the National Research Foundation of Korea (NRF) funded by the Ministry of Education (2017R1D1A1B05028221).”

The original Article has been corrected.

