# Supplementary material for: Qualitative and quantitative evaluation of diabetic choroidopathy using ultra-widefield indocyanine green angiography
Source: Sci Rep. 2023 Feb 13;13:2577. doi: 10.1038/s41598-023-29216-5 (PMC9925819; doi:10.1038/s41598-023-29216-5)
Supplement: Supplementary file 1 — Supplementary Information. [file 41598_2023_29216_MOESM1_ESM.pdf]

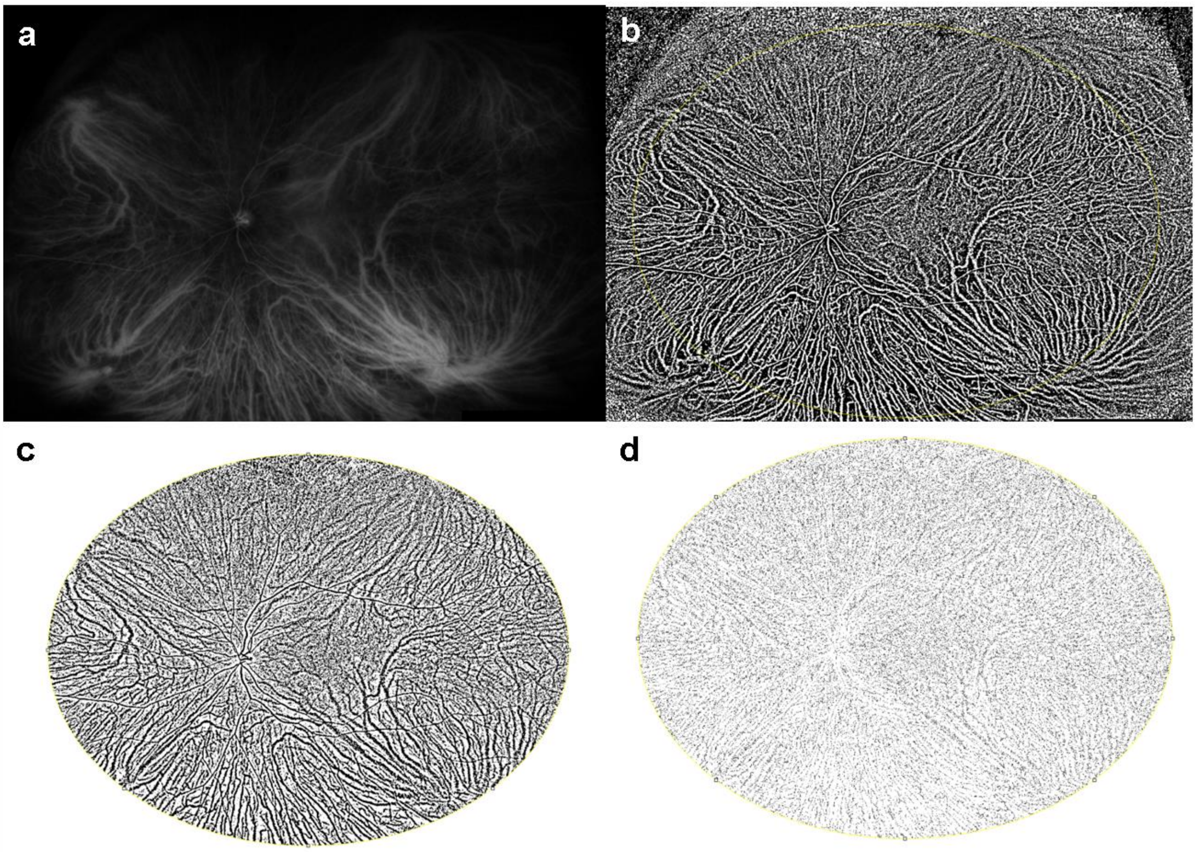

eFigure 1. Quantitative analysis of ultra-widefield indocyanine green angiography (UWF ICGA). The upper row presents the choroidal vascular density (CVD) measure, and the lower row presents the choroidal vascular fractal dimension (CFD) measure. (a) An early phase UWF ICGA image (1 to 2 min after dye injection). (b) An oval region of interest (ROI) (3200 x 2400 pixel) centered on the fovea was determined using the ImageJ oval ROI tool, version 1.53h (National Institute of Health, Bethesda, MD, USA). The binarized image was then adjusted by Niblack's auto local thresholding and converted to an RGB (red, green, and blue) image, and the luminal area was estimated using the color threshold tool. The light pixels were defined as the luminal area, which was automatically calculated by pixel. (c) For CFD calculation, the same ROI, Niblack's auto local thresholding was used, and the binarized image was inverted from white to black and black to white. (d) The inverted image was skeletonized and the CFD was assessed using ImageJ's FracLac plug-in (FracLac 2015Sep090313a9330).
